# Supplementary material for: Development of the Biofidelic Instrumented Neck Surrogate (BINS) with Tunable Stiffness and Embedded Kinematic Sensors for Application in Static Tests and Low-Energy Impacts
Source: Sensors (Basel). 2025 Aug 9;25(16):4925. doi: 10.3390/s25164925 (PMC12390130; doi:10.3390/s25164925)
Supplement: Supplementary file 1 [file sensors-25-04925-s001.zip › sensors-3775602-supplementary.pdf]

Supplementary Table

IC Turns	Front ORings	Back ORings	Lateral ORings (pairs)	Bending Stiffness (Nm/rad)
0	2	0	0	0.22
0	0	2	1	0.17
0	1	1	1	0.23
0	1	1	0	0.21
0	0	0	0	0.28
3	2	0	2	0.19
3	0	2	0	0.15
3	0	1	2	0.19
3	2	1	0	0.19
3	1	2	2	0.14
0	2	2	2	0.14
3	2	2	1	0.14
3	1	0	1	0.21
3	0	0	1	0.24
3	1	0	0	0.22
3	2	1	1	0.17
3	0	0	2	0.22
3	1	1	2	0.18
0	0	2	2	0.17
0	2	2	0	0.16
0	1	0	2	0.21
0	2	0	1	0.20
0	1	2	1	0.17
0	2	1	2	0.15
0	0	1	0	0.23
0	0	0	2	0.23
0	0	0	0	0.30
3	2	1	1	0.16
3	1	1	1	0.19
3	1	1	0	0.19
3	0	0	0	0.23
6	0	0	0	0.17

Table S1: Results of the bending tests for each tested configuration. Front ORings are placed in the flexion side, Back ORings in the extension (stretched) side, Lateral ORings are placed in pairs in the transversal direction.
